# Inorganic pyrophosphate generation by transforming growth factor-beta-1 is mainly dependent on ANK induction by Ras/Raf-1/extracellular signal-regulated kinase pathways in chondrocytes

**DOI:** 10.1186/ar2330

**Published:** 2007-11-22

**Authors:** Frederic Cailotto, Arnaud Bianchi, Sylvie Sebillaud, Narayanan Venkatesan, David Moulin, Jean-Yves Jouzeau, Patrick Netter

**Affiliations:** 1 UMR 7561 CNRS-Nancy-Université, Laboratoire de Physiopathologie et Pharmacologie Articulaires (LPPA) and Faculté de Médecine, Avenue de la forêt de Haye, BP184, 54505 Vandœuvre-Lès-Nancy, France

## Abstract

ANK is a multipass transmembrane protein transporter thought to play a role in the export of intracellular inorganic pyrophosphate and so to contribute to the pathophysiology of chondrocalcinosis. As transforming growth factor-beta-1 (TGF-β1) was shown to favor calcium pyrophosphate dihydrate deposition, we investigated the contribution of ANK to the production of extracellular inorganic pyrophosphate (ePPi) by chondrocytes and the signaling pathways involved in the regulation of *Ank *expression by TGF-β1. Chondrocytes were exposed to 10 ng/mL of TGF-β1, and *Ank *expression was measured by quantitative polymerase chain reaction and Western blot. ePPi was quantified in cell supernatants. RNA silencing was used to define the respective roles of *Ank *and *PC-1 *in TGF-β1-induced ePPi generation. Finally, selective kinase inhibitors and dominant-negative/overexpression plasmid strategies were used to explore the contribution of several signaling pathways to *Ank *induction by TGF-β1. TGF-β1 strongly increased *Ank *expression at the mRNA and protein levels, as well as ePPi production. Using small interfering RNA technology, we showed that *Ank *contributed approximately 60% and *PC-1 *nearly 20% to TGF-β1-induced ePPi generation. Induction of *Ank *by TGF-β1 required activation of the extracellular signal-regulated kinase (ERK) pathway but not of p38-mitogen-activated protein kinase or of protein kinase A. In line with the general protein kinase C (PKC) inhibitor calphostin C, Gö6976 (a Ca^2+^-dependent PKC inhibitor) diminished TGF-β1-induced *Ank *expression by 60%, whereas a 10% inhibition was observed with rottlerin (a PKCδ inhibitor). These data suggest a regulatory role for calcium in TGF-β1-induced *Ank *expression. Finally, we demonstrated that the stimulatory effect of TGF-β1 on *Ank *expression was inhibited by the suppression of the Ras/Raf-1 pathway, while being enhanced by their constitutive activation. Transient overexpression of Smad 7, an inhibitory Smad, failed to affect the inducing effect of TGF-β1 on Ank mRNA level. These data show that TGF-β1 increases ePPi levels, mainly by the induction of the *Ank *gene, which requires activation of Ras, Raf-1, ERK, and Ca^2+^-dependent PKC pathways in chondrocytes.

## Introduction

Chondrocalcinosis is a frequent human disease characterized by the deposition of calcium-containing crystals, mostly calcium pyrophosphate dihydrate (CPPD), within joints. CPPD crystals contribute to cartilage destruction by stimulating mitogenesis of synovial cells as well as synthesis and secretion of proteases, prostanoids, and proinflammatory cytokines that are implicated in cartilage matrix degradation [1]. Several forms of chondrocalcinosis have been described, including idiopathic ones, the frequency of which increases with aging, and familial forms. Some forms of familial chondrocalcinosis, typically inherited in an autosomal dominant manner, were reported to be linked to human chromosomes 8q (CCAL1) or 5p (CCAL2) [2]. Complementary genetic studies demonstrated the linkage between familial forms and the *Ank *gene, located on the CCAL 2 locus. More recently, mutations in the 5' untranslated region of Ank mRNA were also correlated with sporadic forms of chondrocalcinosis [3]. Mutations in the *Ank *gene were reported additionally in autosomal dominant craniometaphyseal dysplasia and ankylosing spondylitis [4,5], supporting a key role for the *Ank *gene in the field of mineralizing arthropathy.

It is generally recognized that a local buildup of excess extracellular inorganic pyrophosphate (ePPi), the anionic component of CPPD crystals, supports CPPD formation [6]. Intracellular inorganic pyrophosphate (iPPi) is a by-product of many synthetic intracellular reactions [7], but there is evidence that it is not able to diffuse across healthy cell membranes. As a consequence, ePPi generation by chondrocytes results from its *de novo *synthesis of ePPi by ecto-enzymes and/or from the contribution of a transport system allowing iPPi to reach the extracellular milieu where CPPD deposition takes place. Among ecto-enzymes, the ecto-nucleoside triphosphate pyrophosphohydrolase, also known as PC-1 (or NPP1), which is abundant in cell membrane [8], hydrolyzes extracellular nucleoside triphosphates into their monophosphate esters and ePPi [9]. On the other hand, the ANK protein was recently postulated to play a key role in the transport of iPPi across the cell membrane. ANK is a multipass transmembrane protein thought to serve either as an anion channel or as a regulator of such a channel [10]. Progressive ankylosis in (*ank*/*ank*) mice is an autosomal recessive form of joint destruction characterized by pathological mineralization in the articular surfaces and synovium [11]. This 'loss of function' mutation in the *Ank *gene increased iPPi concentration while reducing ePPi concentration in (*ank*/*ank*) mouse fibroblasts [10], and these alterations were reversed by overexpression of wild-type *Ank*. This correcting effect of *Ank *was blocked by probenecid, a general inhibitor of organic anion transport [10], which was also shown to inhibit transforming growth factor-beta-1 (TGF-β1)-induced inorganic pyrophosphate (PPi) elaboration by chondrocytes [12]. These data indicate an important role for ANK in the regulation of PPi export.

Finally, accumulation of ePPi in the extracellular milieu could also result from its reduced degradation in the pericellular matrix. Therefore, one must keep in mind that alkaline phosphatase (APase), also known as tissue-nonspecific alkaline phosphatase (TNAP), which is very abundant in chondrocytes adjacent to subchondral bone [13], can hydrolize ePPi into two molecules of extracellular inorganic phosphate. CPPD deposition is therefore highly dependent on the interplay among PC-1, ANK, and TNAP, which tightly regulate the balance between ePPi production and ePPi degradation.

TGF-β1 was shown to be the major growth factor that elevated the production of ePPi by normal chondrocytes [6]. Moreover, it was demonstrated that chondrocyte responsiveness to TGF-β1 increased with aging [14] and that TGF-β1 stimulated ePPi production by articular chondrocytes significantly more in old patients than in younger subjects [15]. These effects were closely related to the occurrence of sporadic chondrocalcinosis [16]. Previous studies showed that Ank mRNA level was higher in human chondrocytes exposed to TGF-β1 than in controls [17], as was also the case for murine cartilage and bone [18]. TGF-β1 was shown to induce two major signaling pathways, referred to as TGF-β1 Smad-dependent [19] or Smad-independent [20] signaling, in many cell types. However, intracellular pathways involved in the regulation of the *Ank *gene are not well documented in chondrocytes, and the contribution of ANK relative to other ePPi-regulating proteins remains unclear. Therefore, the identification of the signaling pathway implicated in *Ank *regulation and subsequent ePPi production by TGF-β1 warrants interest and could lead to insights in the therapy of sporadic chondrocalcinosis.

The present work aimed to investigate the molecular mechanisms underlying the induction of the *Ank *gene by TGF-β1 and to evaluate the relative contribution of ANK to ePPi production in chondrocytes. To that end, we characterized the kinetics of expression of *Ank*, *PC-1*, and *TNAP *in response to TGF-β1. Then, using small interfering RNA (siRNA), we evaluated the respective contributions of *Ank *and *PC-1 *in ePPi production. Finally, using selective kinase inhibitors and a dominant-negative/overexpression plasmid strategy, we distinguished the Smad from the non-Smad signaling events downstream of TGF-β1.

## Materials and methods

### Chondrocyte isolation and culture

Normal articular cartilage was obtained from 6-week-old male Wistar rats (130 to 150 g) killed under dissociative anesthesia (ketamine [Rhône-Mérieux, Lyon, France] and acepromazine [Sanofi Santé Animale, Libourne, France]) in accordance with local ethics committee and national animal care guidelines. Articular cartilage pieces were collected aseptically by joint surgery and were dissected from femoral head caps, and chondrocytes were obtained by sequential digestion with pronase and collagenase B (Roche Diagnostics, Meylan, France) as described previously [21]. Cells were washed twice in phosphate-buffered saline (PBS) and cultured to confluence in 75-cm^2 ^flasks at 37°C in a humidified atmosphere containing 5% CO_2_. Cells were maintained in Dulbecco's modified Eagle's medium (DMEM)/F-12 supplemented with L-glutamine (2 mM), gentamicin (50 μg/mL), amphotericin B (0.5 μg/mL), and heat-inactivated fetal calf serum (FCS) (10%) (Invitrogen Corporation, Cergy Pontoise, France). All experiments reported here were performed with first-passage chondrocytes plated at 4 × 10^5 ^cells per well in six-well plates.

### Chemicals

All chemical reagents, including *para*-nitrophenylthymidine 5'-monophosphate (*p*-NPTMP), *para*-nitrophenyl phosphate (*p*-NPP), and *para*-nitrophenol (*p*-NP), were obtained from Sigma-Aldrich (Saint-Quentin Fallavier, France) unless otherwise indicated. Chemical kinase inhibitors were purchased from Calbiochem (now part of EMD Biosciences, Inc., San Diego, CA, USA): RcAMP (a protein kinase A [PKA] inhibitor), calphostin C (a general inhibitor of protein kinase C [PKC]), rottlerin (an inhibitor of PKCδ) or Gö6976 (an inhibitor of Ca^2+^-dependent PKCα and PKCβI isoenzymes), SB203580 (a p38-mitogen-activated protein kinase [MAPK] inhibitor), or PD98059 (a MAPK extracellular signal-regulated kinase [ERK] kinase 1 [MEK-1] inhibitor).

### Study design

First, we controlled the cell phenotype by measuring the expression level of specific chondrocyte markers (type I, II, and X collagens as well as aggrecan) by real-time quantitative polymerase chain reaction (PCR). Second, we studied the kinetics of expression of *Ank*, *PC-1*, and *TNAP *in response to TGF-β1 (PeproTech France, Neuilly-sur-Seine, France) at the mRNA and protein levels. To that end, chondrocytes were incubated for 1, 3, 6, 12, 24, or 48 hours in the presence or absence of 10 ng/mL of TGF-β1 (prepared from a stock solution at 10 μg/mL in 2 mM citric acid containing 2 mg/mL bovine serum albumin) in DMEM/F-12 containing a final concentration of 1% FCS. Third, to investigate the respective contributions of *Ank *and *PC-1 *in ePPi production, we compared the response with TGF-β1 in chondrocytes transfected with 50 nM of siRNA for each gene. Fourth, to investigate signaling pathways implicated in *Ank *induction by TGF-β1, we performed the kinetics of activation (10 to 180 minutes) of PKC, MAPK, and Smad in cells stimulated with 10 ng/mL of TGF-β1. The contribution of each signaling pathway was assessed by pretreating chondrocytes for 1 hour with the following inhibitors before exposure to 10 ng/mL of TGF-β1 (12 hours): 10 μM of RcAMP (PKA), 1 μM of calphostin C, 5 μM of rottlerin or 5 μM of Gö6976 (PKC), 10 μM of SB203580 (p38-MAPK), or 10 μM of PD98059 (MEK-1). These final concentrations were chosen after preliminary experiments that demonstrated that inhibitors were active (Western blot assessing the phosphorylation of the concerned signaling pathway; data not shown) and not cytotoxic in the MTT (3-[4,5-dimethylthiazol-2-yl]-2,5-diphenyltetrazolium bromide) assay (data not shown). For these experiments, all inhibitors (MAPK, PKA, and PKC) were dissolved in dimethyl sulfoxide (0.1% final concentration). In the last set of experiments, *Ank *expression was studied in chondrocytes electroporated (Nucleofector^®^; amaxa AG, Cologne, Germany) with Ras, Raf-1 (wild-type, constitutively active, or dominant-negative forms) (Clontech-Takara Bio Europe, Saint-Germain-en-Laye, France) or wild-type Smad 7 (Addgene plasmid 11733 [22]) overexpressing plasmids before stimulation or not with 10 ng/mL of TGF-β1.

### RNA extraction and reverse transcription-polymerase chain reaction analysis

Total RNA from cultured chondrocytes was isolated by the acidified guanidinium isothiocyanate method, using TRIZOL^® ^reagent (Sigma-Aldrich). Two micrograms of total RNA was reverse-transcribed for 90 minutes at 37°C in a 20-μL reaction mixture containing 2.5 mM dNTPs, 5 μM random hexamer primers, 1.5 mM MgCl_2_, and 200 U Moloney murine leukemia virus reverse transcriptase (Sigma-Aldrich).

### Real-time quantitative polymerase chain reaction

To quantify aggrecan, Ank, L27, PC-1, TNAP, type IA2, II, or X collagens, and S29 mRNA expression, a real-time quantitative PCR was performed using Lightcycler^® ^(Roche Diagnostics) technology. PCR was performed with SYBR green master mix system (Qiagen S.A., Courtaboeuf, France). The gene-specific primer pairs are described in Table 1. Melting curve was performed to determine the melting temperature of each PCR product, as a control of its specificity, and after amplification, the product size was checked on a 2% agarose gel stained with ethidium bromide (0.5 μg/mL). Analyses depended upon a threshold cycle (Ct) that corresponded to the first clearly detectable increase in fluorescence secondary to SYBR green incorporation into double-stranded DNA. Briefly, the Ct was converted into picograms of DNA using calibration curves made of serial dilutions of known amounts of corresponding purified PCR products. Each run included positive standards and negative reaction controls. The mRNA levels of the gene of interest and of the housekeeping gene *S29 *were determined in parallel for each sample, and results were expressed as the ratio of mRNA level of each gene of interest over the *S29 *gene.

**Table 1:** Gene-specific primer pairs used in real-time quantitative polymerase chain reaction

Gene	Sense	Antisense	Amplicon length (base pair)	GenBank accession number
Aggrecan	5'-ACA CCC CTA CCC TTG CTT CT-3'	5'-AAA GTG TCC AAG GCA TCC AC-3'	124	NM_022190
*Ank*	5'-CAA GAG AGA CAG GGC CAA AG-3'	5'-AAG GCA GCG AGA TAC AGG AA-3'	173	NM_053714
*L27*	5'-TCC TGG CTG GAC GCT ACT C-3'	5'-CCA CAG AGT ACC TTG TGG GC-3'	227	NM_022514
*PC-1*	5'-TAT GCC CAA GAA AGG AAT GG-3'	5'-GCA GCT GGT AAG CAC AAT GA-3'	165	NM_053535
*S29*	5'-AAG ATG GGT CAC CAG CAG CTC TAC TG-3'	5'-AGA CGC GGC AAG AGC GAG AA-3'	67	NM_012876
*TNAP*	5'-GAA CGT CAA TTA ACG GCT GA-3'	5'-CAG ATG GGT GGG AAG AGG T-3'	50	NM_013059
Type IA2 collagen	5'-TTG ACC CTA ACC AAG GAT GC-3'	5'-CAC CCC TTC TGC GTT GTA TT-3'	197	NM_053356
Type II collagen	5'-TCC CTC TGG TTC TGA TGG TC-3'	5'-CTC TGT CTC CAG ATG CAC CA-3'	161	NM_012929
Type X collagen	5'-ATA TCC TGG GGA TCC AGG TC-3'	5'-TGG GTC ACC CTT AGA TCC AG-3'	241	AJ131848

TNAP, tissue-nonspecific alkaline phosphatase.

### Western blot analysis

Rat chondrocytes stimulated or not with TGF-β1 were harvested and lysed in 1× Laemmli buffer (2% SDS, 10% glycerol, 5% 2-β mercaptoethanol, 0.002% bromophenol blue, and 125 mM Tris HCl [pH 6.8]). Protein samples were run on SDS-polyacrylamide gels (10%) and transferred onto a polyvinylidene fluoride membrane (Immobilon; Sigma-Aldrich) as previously described [23]. After 1 hour in blocking buffer (Amersham Biosciences, now part of GE Healthcare, Little Chalfont, Buckinghamshire, UK), membranes were incubated overnight at 4°C with primary antibodies. ANK protein level was determined using rabbit antiserum Ab3 (1:5,000), kindly provided by David Kingsley, Stanford University School of Medicine, Stanford, CA, USA. PC-1 protein level was evaluated using antiserum (1:500) (designed by Eurogentec S.A. [Seraing, Belgium] using a keyhole limpet hemocyanin-coupled peptide of the following sequence: NH_2_-Glu-Arg-Asp-Gly-Glu-Gln-Ala-Gly-Gln-Gly-Pro-Arg-His-Gly-Pro-Cys-COOH) and a polyclonal antibody against β-actin (1:4,000) (Sigma-Aldrich). In signaling pathway experiments, incubation was carried out with anti-phospho-ERK 1/2, anti-phospho-p38-MAPK, anti-phospho-pan-PKC, and anti-phospho-Smad 3/1 (each at 1:500) (Cell Signaling Technology, Inc., Danvers, MA, USA) or β-actin (1:4,000). After three washings with Tris-buffered saline (TBS)-Tween, the blot was incubated with an anti-rabbit immunoglobulin G conjugated with horseradish peroxidase (Cell Signaling Technology, Inc.) diluted at 1:2,000 in blocking buffer for 1 hour at room temperature. After four washings with TBS-Tween, protein bands were detected by chemiluminescence with the Phototope Detection system (Cell Signaling Technology, Inc.) according to the manufacturer's recommendations. The band intensities were quantified by densitometry with a computerized image processing system (Geldoc 2000^®^; Bio-Rad Laboratories, Inc., Hercules, CA, USA).

### Nucleotide pyrophosphatase phosphodiesterase and alkaline phosphatase activity

Rat chondrocytes stimulated or not with TGF-β1 were harvested and lysed in a buffer containing 1% triton X-100, 1.6 mM MgCl_2_, and 0.2 M Tris Base (pH 8.1). Total protein extract (quantified by bicinchonic acid assay) was incubated for 15 minutes with 1 μmol of *p*-NPTMP for nucleotide pyrophosphatase phosphodiesterase (NPPase) activity or for 2.5 hours with 5 μmol of *p*-NPP for APase activity; these enzymatic activities both generate *p*-NP. At the end of incubation, the reaction was stopped by adding exactly 10 μmol of EDTA (ethylenediaminetetraacetic acid) and 200 μmol of NaOH and the absorbance was read at 410 nm. The standard concentrations, ranging from 0 to 0.2 mM *p*-NP, were included in each assay. Results were expressed as units per milligram of total cell proteins, which were quantified by bicinchonic acid assay [24].

### Radiometric assay for extracellular inorganic pyrophosphate

ePPi levels were measured using the differential adsorption of UDP-(6-^3^H) glucose (GE Healthcare), and its reaction product 6-phospho-(6-^3^H) gluconate on activated charcoal, as previously described [25]. The standard concentrations, ranging from 10 to 400 pmol of PPi, were included in each assay. After adsorption of the reaction mixture on charcoal, and centrifugation at 14,000 rpm for 10 minutes, 100 μL of the supernatant was removed carefully and counted for radioactivity in 5 mL of Bio-Safe II (Research Products International Corp, Mt. Prospect, IL, USA). Results were expressed as picomole of ePPi per microgram of total cell proteins.

### Silencing experiments with small interfering RNA

siRNA sequences (designed by Eurogentec S.A.) were Ank sense 5'-CUGGCCAACACGAACAACA-3' and antisense 5'-UGUUGUUCGUGUUGGCCAG-3' and PC-1 sense 5'-GAGGAUGUUUACUCUAUGA-3' and antisense 5'-UCAUAGAGUAAACAUCCUC-3' and were used at final concentrations of 50 nM. Transfections were carried out with one or the other siRNA using X-treme reagent (Roche Diagnostics). Briefly, siRNA and X-treme reagent were diluted separately in serum-free medium, and then diluted X-treme reagent was added to siRNA. After a short incubation at room temperature, cells were washed with PBS and incubated for 4 hours with siRNA-X-treme mix. After this time, medium containing 2% FCS was added to this mix. Stimulations with TGF-β1 (10 ng/mL) were performed the following day. L27 mRNA expression was used as a negative control to check for the specificity of siRNA effects.

### Plasmid electroporation

Chondrocytes were electroporated with plasmids encoding for Ras, Raf-1 (wild-type, constitutively active, or dominant-negative forms) or wild-type Smad 7 (overexpressing plasmid) using Human Chondrocyte Nucleofector^® ^Kit (amaxa AG) according to the manufacturer's protocol. Briefly, trypsinized chondrocytes (1 × 10^6 ^cells) were gently mixed with 6 μg of either plasmid and then were electroporated using Nucleofector^® ^program U-28. Immediately after transfection, cells were split equally into three wells containing 20% FCS-DMEM/F-12 culture medium and were left to recover for 24 hours. Cells were then stimulated or not with 10 ng/mL of TGF-β1 for 12 hours before mRNA (Ank, aggrecan) or 15 minutes before protein (ERK 1/2) extraction. Plasmid pmaxGFP™ (amaxa AG), encoding a green fluorescent protein, was used to determine the transfection efficiency.

### Statistical analysis

Results were expressed as the mean ± standard deviation of at least three independent assays. Comparisons were made by analysis of variance, followed by Fisher *t post hoc *test using Statview™ 5.0 software (SAS Institute Inc., Cary, NC, USA). A *p *value of less than 0.05 was considered significant.

## Results

### Effect of TGF-β1 on the kinetics of proteins regulating inorganic pyrophosphate metabolism in chondrocytes

**Figure 1:**
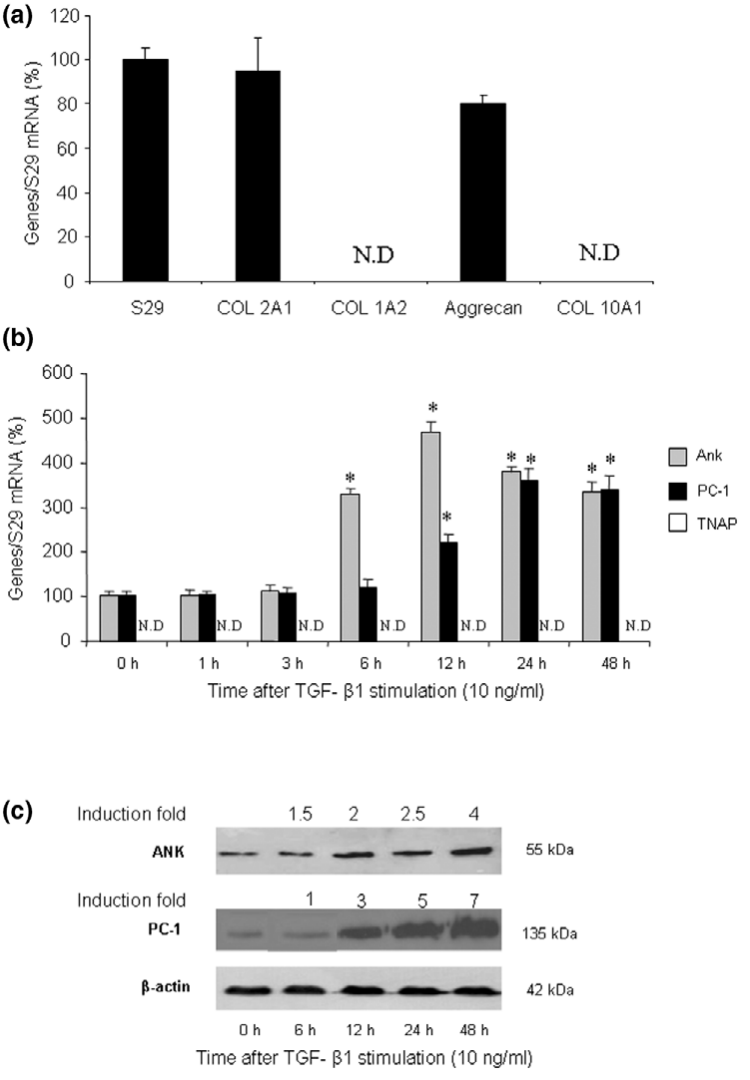
Effect of transforming growth factor-beta-1 (TGF-β1) on proteins regulating inorganic pyrophosphate metabolism. **(a) **Phenotypic characterization of chondrocytes. Total RNA was extracted from untreated rat chondrocytes and subjected to real-time polymerase chain reaction (PCR) analysis. The relative abundance of gene mRNAs was normalized to that of S29 mRNA. Results are presented in histograms as mean percentages (± standard deviation [SD]) over S29 value. **(b) **Effect of TGF-β1 on Ank, PC-1, and TNAP mRNA levels. Total RNA was extracted from rat chondrocytes exposed to 10 ng/mL of TGF-β1 from 1 to 48 hours and subjected to real-time PCR analysis. The relative abundance of gene mRNAs was normalized to that of S29 mRNA. Results are expressed as mean percentages (± SD) over control values. Statistically significant differences from the control are indicated as *p < 0.05. **(c) **Effect of TGF-β1 on ANK or PC-1 protein levels. Total proteins were extracted from rat chondrocytes exposed to 10 ng/mL of TGF-β1 from 6 to 48 hours and subjected to Western blotting using polyclonal anti-ANK and anti-PC-1 antibody. The protein band intensities were quantified by densitometry from enhanced chemiluminescence immunoblots. The relative abundance of these proteins was normalized to that of β-actin protein and expressed as induction folds over control value. N.D., not detected; TNAP, tissue-nonspecific alkaline phosphatase.

Our preliminary experiments confirmed that chondrocytes strongly expressed the cartilage-specific markers aggrecan and type II collagen, whereas the expression of type IA2 and X collagens was not detected (Figure 1a). This confirmed the mature phenotype of the articular chondrocytes used throughout the study, all the more so considering that experiments were carried out with first-passage cells.

Then, we examined the time course of Ank, PC-1, and TNAP mRNA expression in TGF-β1-stimulated chondrocytes (Figure 1b). Ank was upregulated from 6 hours and reached a peak value of 4.5-fold at 12 hours after TGF-β1 exposure. In these conditions, PC-1 was induced approximately 2-fold after 12 hours and reached a maximal induction of approximately 3.5-fold after 24 hours of stimulation with TGF-β1. In contrast, we failed to detect any expression of TNAP in resting chondrocytes or after stimulation with TGF-β1.

Western blotting indicated that ANK protein was induced from 6 hours after TGF-β1 challenge, whereas PC-1 was upregulated after 12 hours (Figure 1c). Taken together, these data demonstrated that Ank was induced by TGF-β1 more rapidly than PC-1 in chondrocytes.

### Effect of TGF-β1 on extracellular inorganic pyrophosphate

**Figure 2:**
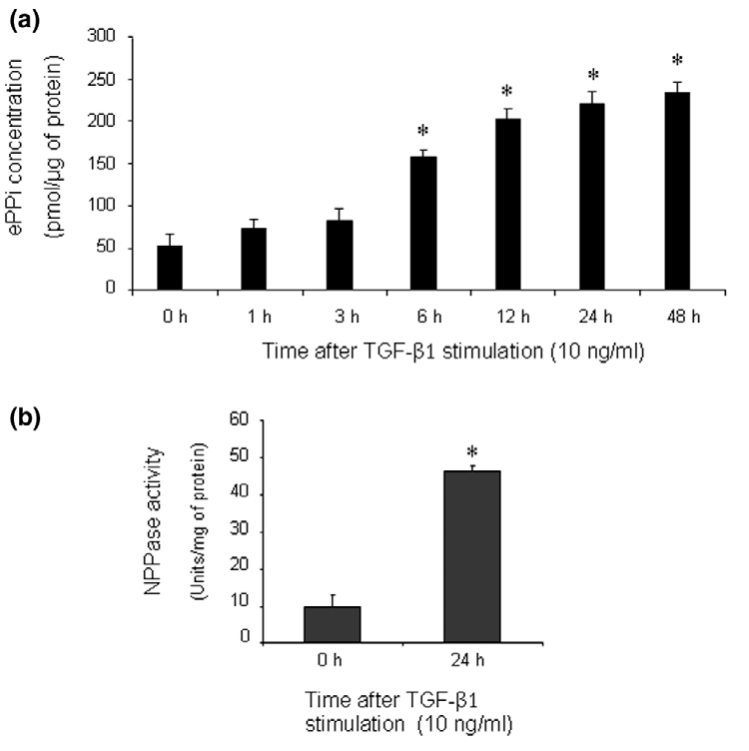
Effect of transforming growth factor-beta-1 (TGF-β1) on extracellular inorganic pyrophosphate (ePPi) and nucleotide pyrophosphatase phosphodiesterase (NPPase) activity. **(a) **Kinetics of ePPi levels in culture supernatant of rat chondrocytes stimulated with TGF-β1 (10 ng/mL). ePPi was assayed radiometrically and normalized to the amount of total cell proteins (*n *= 6). Data are expressed as mean (± standard deviation [SD]) in picomoles per microgram of protein. **(b) **NPPase activity in cultured rat chondrocytes stimulated with 10 ng/mL of TGF-β1. Enzyme activity was normalized to the amount of total cell proteins (*n *= 3). Results are expressed as mean (± SD) in micromoles of paranitrophenol per minute per milligram of protein. Statistically significant differences from the control are indicated as *p < 0.05.

As shown in Figure 2a, ePPi level increased by 3-fold after 6 hours of stimulation with TGF-β1 and reached a plateau from 24 hours (5-fold). In these experimental conditions, TGF-β1 stimulated NPPase activity by 5-fold at 24 hours (Figure 2b). However, we failed to detect any APase activity in our experimental conditions, which is consistent with the lack of expression of type X collagen. These data suggested that TGF-β1 increased ePPi levels by activating ecto-enzymes and not by reducing TNAP activity, which remained undetectable.

### Respective contributions of *Ank *and *PC-1 *to TGF-β1-induced increase in extracellular inorganic pyrophosphate levels in chondrocytes

**Figure 3:**
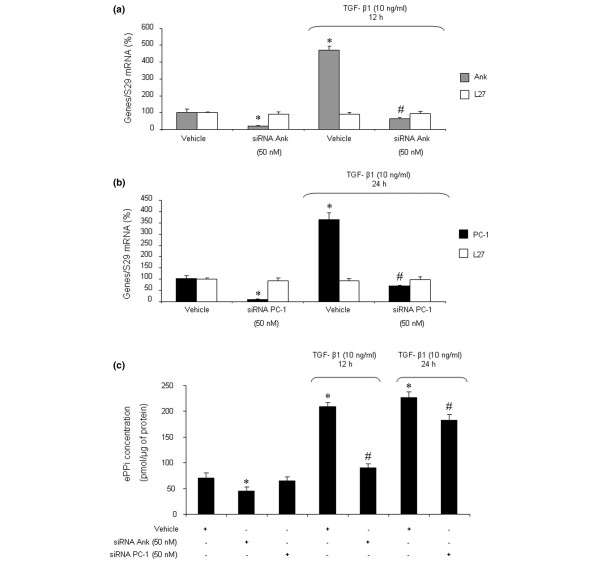
Respective contributions of *Ank *and *PC-1 *to transforming growth factor-beta-1 (TGF-β1)-induced increase in extracellular inorganic pyrophosphate (ePPi) production. Effect of small interfering RNA (siRNA) on Ank **(a) **and PC-1 **(b) **mRNA levels. Rat chondrocytes were transfected with siRNA 24 hours before TGF-β1 stimulation. Total RNA was extracted from rat chondrocytes exposed to 10 ng/mL of TGF-β1 for 12 hours (Ank) **(a) **or 24 hours (PC-1) **(b) **and then subjected to real-time polymerase chain reaction analysis. The level of Ank, PC-1, and L27 mRNAs was normalized to that of S29 mRNA and expressed as mean percentages (± SD) over control values. **(c) **Effect of Ank or PC-1 siRNA on ePPi levels. Shown are levels of ePPi in culture supernatant of rat chondrocytes transfected with siRNA and then stimulated for 12 or 24 hours with 10 ng/mL of TGF-β1. ePPi levels were normalized to the amount of total cell proteins (*n *= 6) and are expressed as mean (± SD) in picomoles per microgram of protein. Statistically significant differences from the control are indicated as *p < 0.05 and from TGF-β1-treated cells as #*p *< 0.05.

The siRNA technology was used to investigate the respective contributions of *Ank *and *PC-1 *on TGF-β1-induced production of ePPi. Control experiments showed that siRNAs were efficient, as they reduced mRNA level by more than 80% in basal conditions (Figure 3a,b) and diminished the stimulating effect of TGF-β1 below control level (Figure 3a,b) at the time of maximal gene expression. No effect was observed on L27 mRNA level in either condition (Figure 3a,b), confirming that the concentration of 50 nM of siRNA was gene-specific for chondrocytes. When ePPi levels were measured in culture supernatant of chondrocytes transfected with siRNA, inhibition of *PC-1 *was ineffective on basal ePPi level and accounted for only a 16% decrease in ePPi level in TGF-β1-stimulated cells (Figure 3c). In contrast, transfection with Ank siRNA reduced the basal ePPi level by 33% and reduced the ePPi level by 60% in TGF-β1-stimulated cells (Figure 3c). These data demonstrated that *Ank *played a major role compared with *PC-1 *in the regulation of ePPi level in resting and TGF-β1-stimulated chondrocytes.

### Identification of TGF-β1-induced signaling pathways contributing to the regulation of *Ank *gene

**Figure 4:**
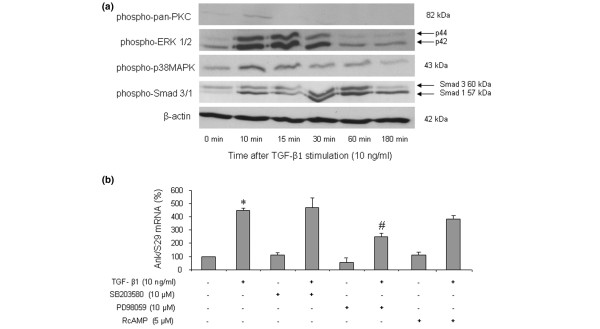
Identification of several signaling pathways in transforming growth factor-beta-1 (TGF-β1)-induced expression of the *Ank *gene. **(a) **Kinetics of signaling events induced by TGF-β1. Total proteins were extracted from rat chondrocytes exposed to 10 ng/mL of TGF-β1 for 10 to 180 minutes and subjected to Western blotting using anti-phospho-ERK 1/2, anti-phospho-p38-MAPK, anti-phospho-pan-PKC, or anti-phospho-Smad 3/1. The relative abundance of these proteins was normalized to that of β-actin protein. **(b) **Effect of specific signaling inhibitors on TGF-β1-induced expression of Ank mRNA. Total RNA was extracted from rat chondrocytes stimulated with 10 ng/mL of TGF-β1 in the presence of 10 μM RcAMP (PKA inhibitor) or 10 μM SB203580 (a selective p38-MAPK inhibitor) or 10 μM PD98059 (a MEK-1 inhibitor) added 1 hour before TGF-β1. The mRNA level of Ank obtained from real-time polymerase chain reaction analysis was normalized to that of S29 mRNA and is expressed as mean percentages (± standard deviation) over control values from three independent experiments. Statistically significant differences from the control are indicated as *p < 0.05 and from TGF-β1-treated cells as #*p *< 0.05. ERK, extracellular signal-regulated kinase; MEK-1, mitogen-activated protein kinase/extracellular signal-regulated kinase kinase 1; p38-MAPK, p38-mitogen-activated protein kinase; PKA, protein kinase A; PKC, protein kinase C.

As shown in Figure 4a, Western blotting revealed that TGF-β1 induced the phosphorylation of p38-MAPK and PKC as early as 10 minutes after stimulation. A major activation of ERK 1/2 was also observed from 10 to 15 minutes after TGF-β1 challenge, whereas a strong phosphorylation of Smad 3 was noted at 30 and 60 minutes. In our experimental conditions, the corresponding non-phosphorylated proteins remained unchanged (data not shown).

When chondrocytes were stimulated by TGF-β1 in the presence of SB203580, a selective p38-MAPK inhibitor, the increase in Ank mRNA level was not affected (Figure 4b). In contrast, PD98059, a selective MEK-1 inhibitor, reduced the stimulatory effect of TGF-β1 by 60% (Figure 4b), supporting a contribution of this MAPK to the induction of the *Ank *gene. RcAMP, a selective PKA inhibitor, was also ineffective in these experimental conditions (Figure 4b). These data showed that TGF-β1 induced multiple signaling pathways in chondrocytes but that neither p38-MAPK nor PKA contributed to its stimulating effect on the *Ank *gene.

### Contribution of protein kinase C pathway to TGF-β1-induced *Ank *expression

**Figure 5:**
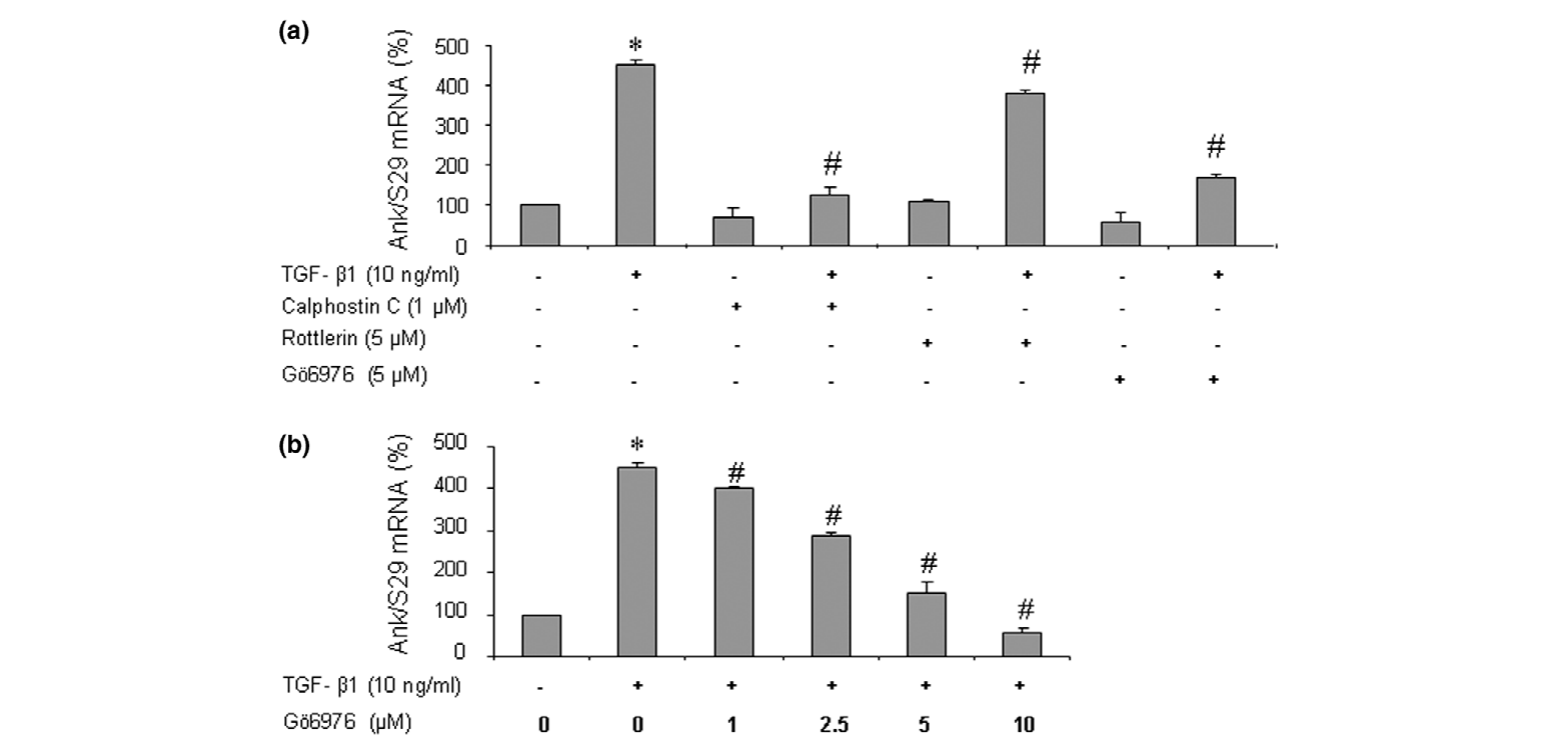
Contribution of protein kinase C (PKC) pathway to transforming growth factor-beta-1 (TGF-β1)-induced expression of the *Ank *gene. **(a) **Effect of PKC inhibitors on *Ank *expression. Total RNA was extracted from rat chondrocytes stimulated with 10 ng/mL of TGF-β1 in the presence of 10 μM of calphostin C (general PKC inhibitor) or 5 μM of rottlerin (PKCδ inhibitor) or 5 μM of Gö6976 (PKC α/β1 inhibitor) added 1 hour before TGF-β1. **(b) **Dose-response study of PKC-dependent induction of *Ank *by TGF-β1. Total RNA was extracted from rat chondrocytes stimulated with 10 ng/mL of TGF-β1 in the presence of 0, 1, 2.5, 5, or 10 μM of Gö6976 added 1 hour before TGF-β1. The mRNA level of Ank obtained from real-time polymerase chain reaction analysis was normalized to that of S29 mRNA and is expressed as mean percentages (± standard deviation) over control values from three independent experiments. Statistically significant differences from the control are indicated as *p < 0.05 and from TGF-β1-treated cells as #*p *< 0.05.

As shown in Figure 5a, the stimulatory effect of TGF-β1 on *Ank *expression was suppressed almost completely by calphostin, a general PKC inhibitor. In these conditions, rottlerin, a specific inhibitor of PKCδ, decreased *Ank *induction by 10% whereas Gö6976, a specific inhibitor of Ca^2+^-dependent PKCs (PKCα and PKCβI), had a 60% inhibitory effect (Figure 5a). A subsequent dose-ranging study showed that inhibition of TGF-β1-induced expression of *Ank *by Gö6976 was clearly dose-dependent and reached the control level at 10 μM (Figure 5b). Taken together, these data supported a major role for PKCα and PKCβI isoenzymes in TGF-β1-stimulated expression of *Ank*.

### Contribution of Ras and Raf-1 pathways to TGF-β1-induced *Ank *expression

**Figure 6:**
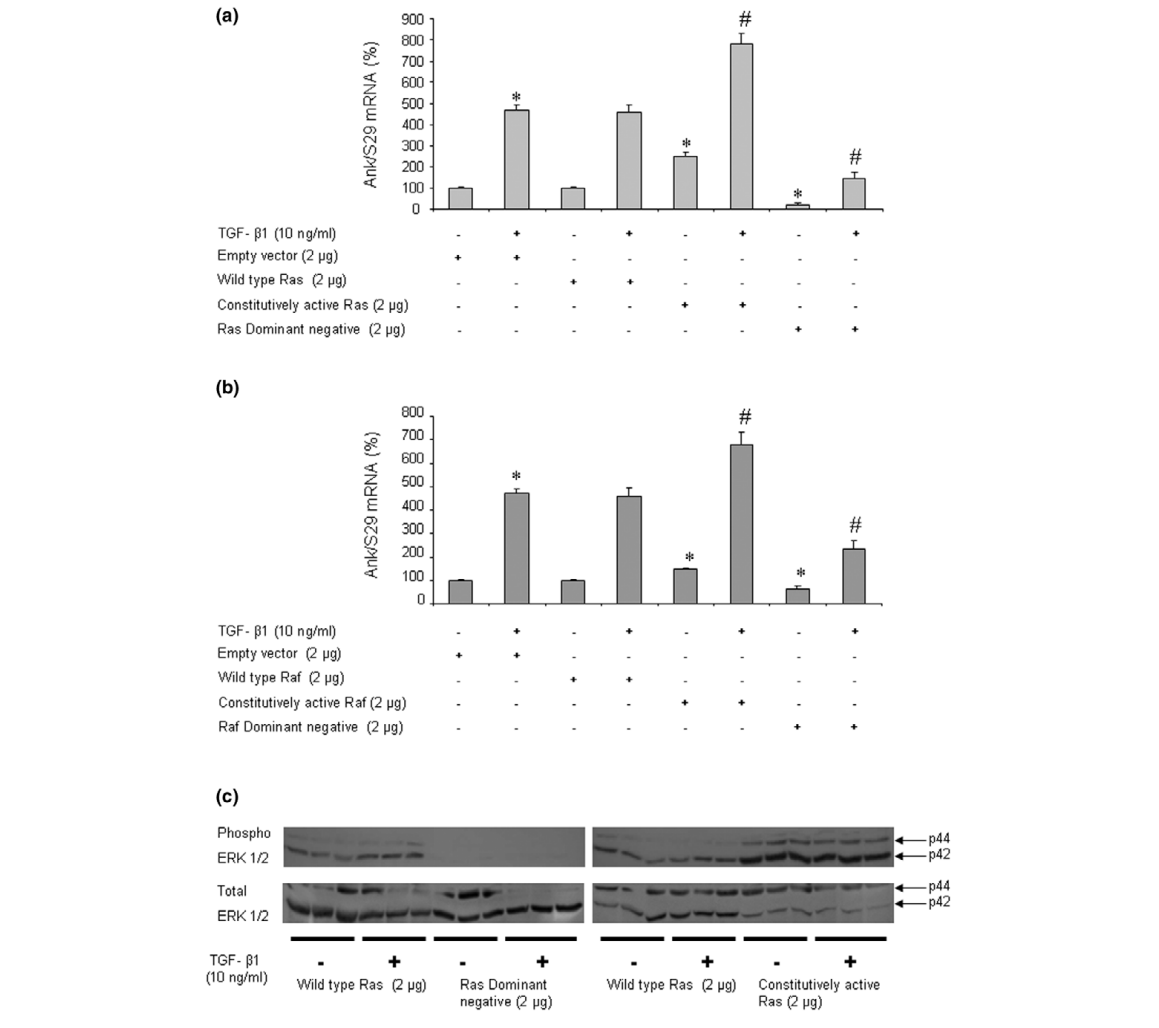
Effect of Ras/Raf-1 modulation on transforming growth factor-beta-1 (TGF-β1)-induced expression of the *Ank *gene. Rat chondrocytes were electroporated with empty vector, wild-type, constitutively active, or dominant-negative plasmids for Ras **(a) **or Raf-1 **(b) **(2 μg/well of six-well plate) before stimulation with 10 ng/mL of TGF-β1 for 12 hours. Total RNA was extracted and subjected to real-time polymerase chain reaction analysis. The mRNA level of Ank was normalized to that of S29 mRNA and is expressed as mean percentages (± standard deviation) over control values from three independent experiments. Statistically significant differences from the control are indicated as *p < 0.05 and from TGF-β1-treated cells as #*p *< 0.05. **(c) **Effect of TGF-β1 on extracellular signal-regulated kinase (ERK) 1/2 phosphorylation in electroporated cells with wild-type, constitutively active, or dominant-negative plasmids for Ras (2 μg/well of six-well plate). Total proteins were extracted from rat chondrocytes exposed to 10 ng/mL of TGF-β1 for 15 minutes and subjected to Western blotting using anti-phospho- and anti-total-ERK 1/2 antibodies. The relative abundance of these proteins was normalized to that of β-actin protein.

The experiments evaluating transfection efficiency did not show any significant difference among the wild-type, the constitutively active, or the dominant-negative forms of Ras and Raf-1 (data not shown). When chondrocytes were transfected to overexpress constitutively active Ras (Figure 6a) or Raf-1 (Figure 6b), the basal expression of *Ank *was increased by 2-fold and 1.5-fold, respectively. In these conditions, the induction of *Ank *by TGF-β1 was increased by 3-fold (Figure 6a) and 4.3-fold, respectively (Figure 6b), which remained similar to cells transfected either with an empty vector or with wild-type Ras or Raf-1 (Figure 6a,b). Transfection with dominant-negative forms of Ras (Figure 6a) or Raf-1 (Figure 6b) reduced both basal (by 75% and 45%, respectively) and TGF-β1-induced (by 3-fold and 2-fold, respectively) *Ank *expression. These data supported a major role for the Ras/Raf-1 pathway in TGF-β1-induced expression of *Ank*.

Western blot analysis of ERK 1/2 phosphoproteins showed an activation of ERK 1/2 pathway by TGF-β1 in cells transfected with wild-type Ras (Figure 6c). More importantly, the phosphorylation of ERK 1/2 was suppressed almost completely in cells electroporated with a dominant-negative form of Ras, whereas overexpression of constitutively active Ras raised both basal and TGF-β1-induced levels of ERK 1/2 phosphorylation (Figure 6c). These results, similar to those obtained with equivalent Raf-1 constructs, demonstrated that activation of ERK 1/2 by TGF-β1 involved the Ras/Raf-1 pathway in chondrocytes.

### Induction of *Ank *expression by TGF-β1 is a Smad-independent event

**Figure 7:**
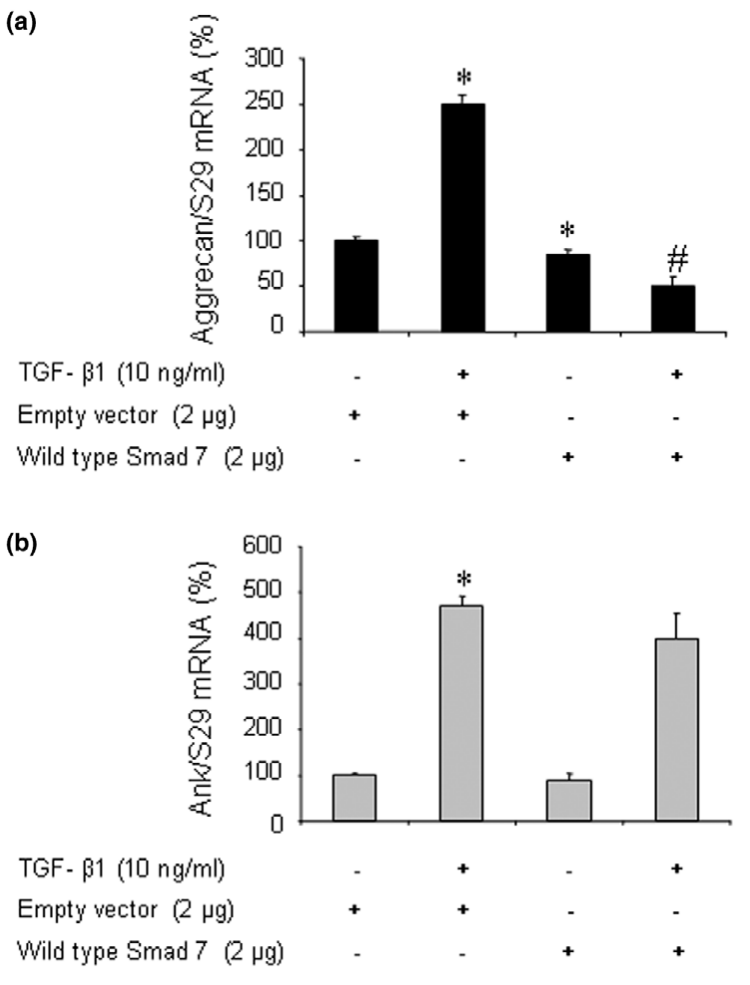
Effect of Smad 7 overexpression on transforming growth factor-beta-1 (TGF-β1)-induced responses in rat chondrocytes. Rat chondrocytes were electroporated with either empty vector or wild-type Smad 7 overexpressing plasmid (2 μg/well of six-well plate) and then treated for 12 hours with 10 ng/mL of TGF-β1. Total RNA was extracted and subjected to real-time polymerase chain reaction analysis. The mRNA level of aggrecan **(a) **and Ank **(b) **was normalized to that of S29 mRNA and is expressed as mean percentages (± standard deviation) over control values from three independent experiments. Statistically significant differences from the control are indicated as *p < 0.05 and from TGF-β1-treated cells as #*p *< 0.05.

In chondrocytes transfected to overexpress wild-type Smad 7, an inhibitory Smad, control experiments showed that the basal expression of aggrecan, chosen as a specific Smad-dependent gene in chondrocytes, was reduced marginally (Figure 7a). In contrast, the stimulating effect of TGF-β1 on aggrecan was abolished, thus demonstrating the efficiency of the construct (Figure 7a). However, neither the basal expression of *Ank *nor its induction by TGF-β1 was affected by overexpression of Smad 7 in chondrocytes (Figure 7b). These data demonstrated that the Smad pathway did not play a major role in the induction of the *Ank *gene by TGF-β1.

## Discussion

Previous studies demonstrated a major contribution of ANK in the regulation of ePPi levels. ANK is a transporter able to export iPPi from the cells and is known to be upregulated in osteoarthritis [17,26]. Moreover, chondrocytes and cartilage extracts from patients with CPPD disease express high levels of Ank mRNA [17]. Besides, chondrocytes treated with TGF-β1 generated more ePPi than normal chondrocytes [15], and chondrocyte sensitivity to TGF-β1 increased with aging [14]. These data led us to suppose that *Ank *could be a major target of TGF-β1 in chondrocytes, likely contributing to its pathophysiological relevance to CPPD deposition.

In our experimental conditions, TGF-β1 increased both Ank mRNA and ANK protein levels, this induction beginning as early as 6 hours. PC-1 mRNA was also induced by TGF-β1 but in a more delayed fashion, whereas expression of TNAP could not be detected. Our data confirmed that TGF-β1 stimulated ePPi production [15] and demonstrated that this was concomitant with the increased expression of *PC-1 *and *Ank*. Moreover, similar experiments performed on cartilage explants showed an identical pattern of response, thus validating our monolayer culture system of chondrocytes (F. Cailotto, A. Bianchi, S. Sebillaud, N. Venkatesan, D. Moulin, J-Y. Jouzeau, P. Netter, unpublished data). Based on the higher levels of TGF-β1 found in the synovial fluid of osteoarthritis patients having developed CPPD deposition [27], our findings are in favor of a pathophysiological contribution of TGF-β1-induced dysregulation of *Ank *and *PC-1 *in sporadic chondrocalcinosis.

To date, no data are available on the relative contributions of ANK and PC-1 to TGF-β1-induced changes in ePPi production. Therefore, we developed an siRNA technology to clarify the respective roles of ANK and PC-1 since both genes differed rather in their kinetics of expression than in their extent of induction in response to TGF-β1. We demonstrated that, for a comparable knockdown of target genes by siRNA, *Ank *contributed 4-fold more than *PC-1 *to TGF-β1-induced increase in ePPi level. The minor effect of PC-1 siRNA on ePPi level could possibly be explained by a stronger basal expression of PC-1 (and, consequently, a residual enzymatic activity even after siRNA transfection) compared with ANK in our cell culture system. However, our data were not biased by the kinetics of induction of each gene since the consequence of their knockdown was studied at the time of their maximal expression in response to TGF-β1. Moreover, we also observed that Ank siRNA diminished TGF-β1-induced ePPi production by 65% at 24 hours (data not shown), confirming that the contribution of *Ank *was still the most significant at the time of maximal expression of PC-1 mRNA. Our results could be partly explained by the fact that ANK is a multipass transmembrane protein thought to serve either as an anion channel or as a regulator of such a channel [10]. As a consequence, an increase or a decrease of protein level could have a profound and rapid impact on PPi transport across the membrane. PC-1 has been shown to be strongly induced by TGF-β1, as well as NPPase activity in chondrocytes [28], and our data confirmed these observations. However, the contribution of PC-1 to ePPi generation could be estimated to be between 35% to 50% in osteoblasts [29], suggesting that PC-1 is an important contributor but not the major contributor of ePPi generation, which is consistent with our findings in chondrocytes. One important consideration is that, even though knockdown with siRNA was very efficient at the mRNA level, neither the repression of *Ank *nor that of *PC-1 *was sufficient to diminish ePPi levels below control level in TGF-β1-stimulated cells. This suggests that, although *Ank *seemed to play a major role, the generation of ePPi could require a coordinated contribution of *Ank *and *PC-1*, as suggested by others [26].

When we studied the regulation of *Ank *expression by TGF-β1, we demonstrated firstly that ERK 1/2 and p38-MAPKs were activated in our cell culture system. These results agree well with their contribution to the inducing effect of TGF-β1 on aggrecan [30] or TIMP-3 [31] expression in chondrogenic cells. Complementary experiments with specific kinase inhibitors showed that inhibition of MEK-1 by PD98059 reduced the stimulating effect of TGF-β1 by 2-fold. In contrast, the lack of efficiency of SB203580 demonstrated that p38-MAPK was not involved in TGF-β1-induced expression of *Ank*. Our results are in accordance with other studies reporting that the contribution of the p38-MAPK pathway to the TGF-β1 effect in cartilage was influenced greatly by the species [32] and cell culture system [33].

To investigate further the contribution of the MEK-1 pathway, we modulated the expression of Ras and Raf-1 proteins, which are known to trigger MEK-1 activation in chondrocytes [34]. Our results showed that Ras and, to a lesser extent, Raf-1 were implicated in TGF-β1-induced expression of *Ank *in chondrocytes, a finding consistent with data demonstrating the activation of Ras and Raf-1 by TGF-β1 in several cell systems [35,36].

Furthermore, we demonstrated that the activation of ERK 1/2 by TGF-β1 was strongly dependent on the activation of Ras and Raf-1 since transfection with dominant-negative forms of these proteins completely inhibited the stimulating effect of TGF-β1. Moreover, constitutively active forms raised both basal and TGF-β1-induced ERK 1/2 phosphorylation. Taken together, these findings confirmed the linkage between activation of Ras/Raf-1, MEK-1, and ERK pathways [34-36] and highlighted their contribution to the induction of *Ank *by TGF-β1 in chondrocytes.

Ryan and colleagues [37] demonstrated that, in addition to MAPKs, adenylate cyclase activation and generation of cAMP reduced ePPi production in chondrocytes. The PKA pathway was also described to be induced by TGF-β1 in chondrocytes [38,39]. However, using RcAMP as a PKA inhibitor, we failed to demonstrate any contribution of PKA to the induction of *Ank *by TGF-β1.

In chondrocytes, the production of ePPi was also reported to be stimulated by PKC-dependent pathways [37], which are known to be activated by TGF-β1 [40]. We showed that the stimulation of *Ank *expression depended on PKC activation but with a variable effect of the inhibitors depending on their specificity for PKC isoenzymes. Thus, when cells were pretreated with calphostin C (a broad PKC inhibitor), a strong effect on Ank mRNA expression was observed whereas rottlerin (a PKCδ inhibitor) was weakly effective. These findings suggested that PKCδ, alongside the other members of the novel PKC family (PKCε, PKCη, and PKCθ [41]), was not implicated in the regulation of *Ank *expression by TGF-β1. In contrast, we demonstrated that Gö6976 (a Ca^2+^-dependent PKCα and PKCβI isoenzyme inhibitor) was strongly inhibitory. Since the only difference between novel PKC and conventional PKC (for example, Ca^2+^-dependent PKCα and PKCβI) activation is the dependence on calcium [41], our findings support a possible regulatory role for calcium in the induction of *Ank *expression by TGF-β1.

Some genes induced by TGF-β1 are partly or totally regulated by Smad proteins. We demonstrated that, in our experimental conditions, TGF-β1 was able to induce the phosphorylation of Smad 3, known to modulate the expression of chondrocyte-specific genes, such as aggrecan [42]. Smad 7 is a natural inhibitor of the Smad pathway, preventing Smad 3 phosphorylation in the cytosol and therefore suppressing Smad signaling [19]. Scharstuhl and colleagues [42] demonstrated that Smad 7 overexpression was able to counteract TGF-β1-induced expression of aggrecan in chondrocytes, as was the case in our experiments. However, we demonstrated that overexpression of wild-type Smad 7 failed to reduce TGF-β1-induced expression of *Ank *in chondrocytes, thus demonstrating that it was a non-Smad signaling event.

Taken as a whole, these observations suggest that, contrary to degenerative joint pathologies [43], targeting the non-Smad TGF-β1 signaling events may lead to insights in the field of sporadic chondrocalcinosis since the repression of *Ank *would translate into reduced ePPi levels in synovial fluid. Thus, selective conventional PKC inhibitors should be powerful agents, considering that some of them, such as Gö6976 [44,45], are currently in clinical development for cardiovascular diseases and cancer. Moreover, probenecid was shown to inhibit *Ank *function [10] and TGF-β1-induced PPi elaboration by chondrocytes [12]. Therefore, the effect of the combination of probenecid and Gö6976 on CPPD formation could be worthy of study.

## Conclusion

To summarize, the present study shows that TGF-β1 increases ePPi levels, with a major contribution of *Ank*, despite its similar inducing effect on *Ank *and *PC-1*. These results are in favor of a main role of *Ank *as an effector of TGF-β1 in CPPD formation. Induction of *Ank *is mediated by the dual activation of Ras, Raf-1, and MEK-1/ERK cascade and Ca^2+^-dependent PKC but is independent of the Smad signaling pathway. Our data support the strong contribution of *Ank *to the pathogenesis of sporadic chondrocalcinosis, in addition to familial chondrocalcinosis, and identify signaling pathways involved in TGF-β1-induced *Ank *expression which could lead to new therapies for CPPD disease.

## Abbreviations

APase = alkaline phosphatase; CPPD = calcium pyrophosphate dihydrate; Ct = threshold cycle; DMEM = Dulbecco's modified Eagle's medium; ePPi = extracellular inorganic pyrophosphate; ERK = extracellular signal-regulated kinase; FCS = fetal calf serum; iPPi = intracellular inorganic pyrophosphate; MAPK = mitogen-activated protein kinase; MEK-1 = mitogen-activated protein kinase/extracellular signal-regulated kinase kinase 1; NPPase = nucleotide pyrophosphatase phosphodiesterase; PBS = phosphate-buffered saline; PCR = polymerase chain reaction; PKA = protein kinase A; PKC = protein kinase C; *p*-NP = *para*-nitrophenol; *p*-NPP = *para*-nitrophenyl phosphate; *p*-NPTMP = *para*-nitrophenylthymidine 5'-monophosphate; PPi = inorganic pyrophosphate; siRNA = small interfering RNA; TBS = Tris-buffered saline; TGF-β1 = transforming growth factor-beta-1; TNAP = tissue-nonspecific alkaline phosphatase.

## Competing interests

The authors declare that they have no competing interests.

## Authors' contributions

FC performed cell culture, RNA extraction, real-time quantitative PCR, ePPi assays, and siRNA assays and was involved in drafting the manuscript. AB carried out the inhibitor experiments, drafted the manuscript, and contributed to the study design. SS performed chondrocyte isolation, cell culture, Western blot analysis, and real-time quantitative PCR. NV participated in revising the manuscript and in the interpretation of data. DM carried out RNA extraction and participated in revising the manuscript. J-YJ and PN contributed to the study design and revised the manuscript for intellectual content. All authors read and approved the final manuscript.
